# Forearm muscles fatigue induced by repetitive braking on a motorcycle is best discriminated by specific kinetic parameters

**DOI:** 10.1371/journal.pone.0246242

**Published:** 2021-02-05

**Authors:** Michel Marina, Priscila Torrado, Stéphane Baudry, Jacques Duchateau

**Affiliations:** 1 Research Group in Physical Activity and Health (GRAFiS), Institut National d’Educació Física de Catalunya–University of Barcelona, Barcelona, Spain; 2 School of Health Sciences, TecnoCampus Mataró–Universitat Pompeu Fabra, Mataró, Spain; 3 Laboratory of Applied Biology and Neurophysiology, Université Libre de Bruxelles, Bruxelles, Belgium; Universite de Nantes, FRANCE

## Abstract

Maneuvering a motorcycle in racing conditions or for prolonged time is sufficiently demanding that on many occasions forearm muscles reach a state of functional failure when riders cannot properly brake or operate the throttle. This study intends to discriminate which ones of the several dynamometric parameters used in the literature to characterize the Force-time (F-t) curve during voluntary contractions are more sensitive to neuromuscular fatigue in simulated motorcycle-riding conditions. Thirty-three adults performed an intermittent fatiguing protocol (IFP) that simulated the brake-pulling and throttle-twisting actions, by using a hydraulic system equipped with a pressure sensor. Sixty pressure-time (P-t) curve parameters, including the rate of pressure development (RPD) and area under the curve were measured to characterize the time course of the braking maximal voluntary contraction (MVC). Two types of variables were used to analyze the P-t curve: 1) Times interval (from 0 to 30-50-100-500-1000 and 2000 ms); 2) Percentages of MVC (10-30-60-90%MVC). Overall significant (*p* ≤ 0.05) fatigue-related declines were observed only at time intervals longer than 100 ms and contraction intensities higher than 30%MVC. Strong and significant linear declines (*p* < 0.001) were observed at 500 ms and 1 s for normalized pressures, as well as for the ratio RPD_60%MVC_/MVC (*p* < 0.003) throughout the IFP. Our results suggest considering RPD at time windows of 0–500 ms and 0–1 s, and contraction intensities comprised between 30% and 60% of MVC, as more suitable criteria to study fatigue-related decrements in performance rather than the classical MVC force.

## Introduction

The braking gesture on a motorcycle can be considered as a handgrip task. Because such repetitive movements occur in many contexts and situations such as in music, sport, or industrial work, scientific research studies reproduced them to investigate neuromuscular fatigue during continuous [1–5] and intermittent [2, 6–8] contractions. The interest in handgrip tasks to assess neuromuscular fatigue is widely justified among motorcycle racers because of its association with the prevalence of the forearm chronic exertional compartmental syndrome–CECS- [9–15]. These disorders are related to tasks that involve forceful and repetitive muscular exertions maintained for such a long time that they may lead to significant neuromuscular fatigue and functional impairment [16]. Due to the regulations on public roads and the greater level of safety, the option of attending training sessions at a race track with their own motorcycle is more and more common among riders not oriented to professional racing. In this context of sport riding for fun, a great number of individuals also have similar complaints (heavy forearm feeling and fatigue) at the end of the day.

Previous investigations that focused on motorcycle riders used the decrement in force during a maximal voluntary contraction (MVC) as the first functional manifestation of fatigue [7, 8, 17]. The focus on MVC force decrement is justified because it is a widely accepted method to quantify neuromuscular fatigue [18–22] and its relationship with sport performance in general. However, when racing with a motorcycle, kinetics of the maneuvers is crucial to improve the performance. A representative kinetics parameter is the rate of force development (RFD), which is also affected by fatigue and strongly associated with performance in sports where the ability to make explosive efforts is essential and time to develop force is limited [23, 24]. A more innovative approach reinforces the idea that, as compared to MVC force, RFD seems to be better related to most performances of both sport-specific and functional daily tasks [25, 26]. This rationale can be applied to motorcycle racers, who are required to finely modulate and maintain the braking performance for multiple times and prolonged periods during their competitions [27]. Top-level riders must possess proper levels of physical condition to manage successfully the considerable physical loads (inertial and centrifugal forces) present when racing their motorcycle [28]. The fact that one out of every four braking actions generates inertial stresses greater than 10 m/s^2^ [27, 29] supports the previous remark.

RFD is generally used to describe the ability to generate high muscular force within a brief period of time, and has been generally defined as the slope of the F-t curve (ΔForce/Δtime) [23], determined by the capacity to produce maximal voluntary activation in the early phase of an explosive action [25]. Moreover, its advantage over an MVC, is that RFD is more sensitive in detecting acute and chronic changes in neuromuscular function [30–33], and is potentially governed by different physiological mechanisms in comparison to MVC force [34, 35].

The area under F-t curve has been used as far back as six decades ago [5], to estimate the fatigue-related effects on the shape of force development curves during dynamic handgrip actions. Nevertheless, two studies addressed the changing shape of the F-t curve after a fatiguing task characterized by the maintenance of a maximal handgrip contraction until the force decreased to specific intensities (80–60 and 40%) with respect to the initial MVC [3, 4].

The rising part of the F-t curve, is typically assessed during: a) single joint actions [23, 24, 36] because they allow a better experimental control than multi-joint actions, and b) isometric actions such as changes in muscle length-velocity influence force and the measurement of explosive force production [37]. Nevertheless, sports activities are multiple-joint dynamic actions and thus provide distinct neural and mechanical conditions to those in which explosive force production is typically measured [26]. So, the association between the experimental setup and the braking gesture on a motorcycle facilitates the inference of practical implications. In the interest of reducing the barriers between laboratory research and in-field sport practice, the experimental setup of this study replicates the gesture of riding and braking a motorcycle in static conditions with high fidelity, substantially supporting the practical applications of such research. Indeed, in this study, the direct pressure inside the hydraulic braking system was measured, instead of the force, determining a specific *rate of pressure development* (RPD) and *pressure-time curve* (P-t curve), instead of the classical terms of RFD and F-t curve respectively.

Knowing the difficulties in evaluating the RFD [25] in a valid and reliable way, the main interest of this study was to determine, among a great number of dynamometric parameters used to characterize the P-t curve, the ones that are most sensitive to a state of neuromuscular fatigue induced by repetitive lever-pulling (braking) and handle-twisting (throttle opening) up to exhaustion. The second objective was to investigate the effect of an intermittent fatiguing protocol (IFP) specifically designed for motorcycle riders.

The performance in motorsports is influenced by the ability to perform brief and precise maneuvers (i.e. braking) throughout the entire race [29]. Therefore, studying the changes of the P-t curve during a fatiguing protocol could help to support specific training methods in these sports. With respect of the above-mentioned objectives, we hypothesized that the most sensitive parameters to fatiguing braking actions would be found during an early time-window and at lower contraction intensities of the P-t curve. Our hypothesis is based on the fact that during fast actions, RFD is closely associated with the subject’s capacity to rapidly activate most motor units with very high discharge rate at the onset of the contraction [34, 38–40]. As high-threshold (fast) motor units play a key role in RFD and these units are more prone to fatigue than low-threshold units [25, 41, 42], it is expected that the greater decrements in performance induced by our IFP is located more particularly at the initiation [43] of the braking actions.

## Method

This study was approved by the Clinical Research of the Catalan Sports Council with the approval number 15/2018 CEICGC. Informed consent was obtained from all subjects after a full explanation of the experimental purpose and protocol.

### Subjects

Thirty-three male subjects (aged 28 ± 8 years; stature 176 ± 5 cm; body mass 74 ± 6 kg) volunteered to participate in the study. All of them habitually used a motorcycle in their daily activity or for leisure. Leisure include going to a road race track for fun with their own motorcycle and occasionally attend motorcycle riding classes in “on-track” situations to improve their riding skills and safety margin when using their own motorcycle during daily life. All subjects came twice to perform the same IFP with less than 2 weeks between sessions. The first day served as a familiarization session and the second day to do the test.

### Dynamometric assessment

The static structure used to simulate the position of a rider on a road race motorcycle was substantially improved and upgraded with respect to previous studies [2, 7, 8]. In the present study the replication and analogy regarding the real situation is practically identical because the instrumental setup reproduces the same mechanical conditions, apart from the obvious impossibility of simulating the deceleration of the vehicle. The whole braking system composed by the brake lever, radial master cylinders, metal hoses and the brake liquid, caliper, pads and spacer (in substitution of the disc), was exactly the same as the ones habitually used in road motorcycles for racing or sports use (Brembo S.p.A, Bergamo, Italy). The sensors used to measure the pressure within the hydraulic circuit of the braking system and the length of the throttle rotation (CSR Motorsport, Spain, model SY-KITPBrake-000/ SA-PK100M10 and AC-APS04-000/SA-APS04-000 respectively) were of the same type as the ones used by the racing teams in Grand Prix, Supersport, Superbike and FIM-CEV Repsol International disciplines organized by the “*Fédération Internationale de Motocyclisme* (FIM)”. Concerning the total compliance of the brake lever, it is important to highlight that the first 2.8 cm displacement of the outer tip of the lever required an external force equivalent to 2 bars of pressure inside the hydraulic system.

The participants were asked to exert a force against the brake lever (using only the right hand), that is, with the second and third finger (i.e. index and middle fingers) holding the lever half way, keeping the thumb and the other fingers grasping the throttle of the handlebar [8]. Although this is not the only option, the “two fingers braking technique” is the standard recommendation of the road race motorcycle schools to road riders not oriented to racing but who want to improve their riding skills.

The brake lever was adjusted to each participant’s hand size and a continuous linear feedback was displayed on a 24-inch screen to inform the subjects of the magnitude of the force they exerted against the brake lever, and the magnitude of the throttle-twisting actions. The body posture (elbow, wrist and trunk angles) was checked according to the same criteria used in previous studies [2, 7, 8]. The force exerted against the brake lever was measured with a pressure sensor commonly used in road racing motorcycles. A linear relationship between the raw signal recorded by the brake sensor and the pressure inside the hydraulic circuit of the braking system (5v = 100 bars; multiplication factor = 20) was ensured by the manufacturer. We assume a linear relationship between the pressure measured inside the hydraulic system, and the force exerted against the lever. The analog raw signal [volts] was sampled at 2000 Hz using an external analog-to-digital (A/D) converter (Power 1401; Cambridge Electronics Design [CED], Cambridge, UK) and recorded online with Spike2 software (CED). For the offline analysis, braking raw signal was low pass digital filtered (8 Hz), using a fourth-order Butterworth filter [25, 44].

### Protocol

Three minutes before the initiation of the IFP, the participant had to exert two MVCs brake trials of 3-s duration separated by a 1-min rest. All the MVCs (baseline, IFP and recovery) had a duration of three seconds and were done in a “step and hold” manner, during which each participant was instructed to increase the force as quickly as possible and maintain his maximal force for a few seconds [40]. This procedure, accompanied by instruction of the kind “hard and fast” was more adequate to study the RFD, because it produced the highest forces and greater RFD for 94% of the subjects who had also done the same test but with the instruction of “only fast” [45].

As in other studies [2, 7, 39], the beginning of each “explosive and hold contraction” was indicated by an auditory cue and participants were instructed to avoid any counter-movement or pre-tension. The highest MVC was considered as the baseline value. During the IFP, subjects were asked to perform work/rest cycles of 6 s braking action at 30% MVC, alternating with periods of 4 s, in which they vigorously rotated the gas throttle twice (as when speeding up their motorcycle). The braking intensity of 30% MVC was adopted after consulting with world class professional riders, who coincided about the perception of applying approximately this percentage of force during a very strong braking in real situation [2]. The magnitude and duration (1.5 s) of the two consecutive throttle actions, in between each one of the six lever-pulls (braking actions), were also assisted by an auditory clue very similar to the real situation. That is, the sound of a motor in full acceleration with one gearshift in between. This sequence, which was systematically ended with a MVC of 3-s duration, was considered as a round. A recovery interspace of 5-s duration was included between each round of the IFP. The rounds were repeated until the subjects were unable to maintain two consecutive braking actions, or when their MVC force declined by 10% below the 30% MVC target pressure. In order to facilitate comparisons, independently of the number of rounds executed by each subject, the total number of rounds represented 100% of their performance [46]. To examine the time course parameters of the MVCs’ P-t curves throughout the IFP, the whole performance (100%) was divided into five successive sections called “normalized rounds” (Rn), which corresponded to the mean value of the variable calculated over each 20% time window. Ten minutes after the end of the IFP, a last MVC was performed to check the recovery state of performance.

### Data processing

The onset of contraction was determined manually and always by the same investigator, considering this procedures as gold standard for being more sensitive, valid and accurate [44]. To identify the sudden change from rest to pressure production, the signal was amplified (scale: 0.01 v = 0.002 bar) to verify the time at which pressure deflects away from the baseline (~0.03 bar equivalent of ~0.86% of MVC) [44].

Two types of pressure parameters were used: 1) The raw data (Pressure, P_val_) [bars] to plot the raw Pressure-time curve (P-t curve); and 2) the normalized data (nP_val_) [%] with respect to basal condition. Following recommendations of previous studies [3, 4], both absolute and normalized scores of pressure were analyzed to study the changing shape of the P-t curve due to fatigue.

The parameters used to analyze the P-t curve were chosen according to two different criteria: 1) Specific times interval from the onset of contraction has been adopted by numerous studies [3, 23, 24, 47–52] to infer the relative neural and muscle contributions to the decline in performance during distinct phases of the contraction [25, 39]. Time windows between pressure onset and 30, 50, 100, 500, 1000 and 2000 ms were chosen to analyze the early and late phases of the P-t curve; 2) Selection of specific proportions [50] or percentages of the MVC [3, 5, 49]. After visual inspection of the signal during the pilot phase we adopted the percentages (10, 30, 60, 90% of MVC) proposed by Hakkinen et al. [50]. The 10% MVC was chosen because of the excessive within subject variability (CV > 25%) of the kinematic signal at low contraction intensities (less than 10%) in a non-fatigued state. The other three intensities because of their equidistance across the full range of pressure, and taking into account that 30%MVC was successfully used in previous fatigue related investigations specifically focused on the braking gesture on a motorcycle [2, 7, 8].

Within each time and %MVC windows, the pressure values (Pval) [bars] and the time [sec] to reach a specific percentage of MVC (Time_%MVC_) were measured. The area below the P-t curve, computed as integral time-pressure in the specific time-windows of analysis (Area_ms_) [bar·s], was considered representative of the impulse [23, 24, 39, 48, 49]. The Area [bar·s] was also calculated for each specific %MVC window (Area_%MVC_). The rate of pressure development (RPD) was computed as the slope of the P-t curve (ΔP/Δt) [bar/s] over the different time intervals [23, 25]. According to previous studies [23], RPD was also calculated within the time interval needed to reach a determined portion or percentage of the MVC (RPD_%MVC_). RPD values were normalized to the MVC [39, 49, 53].

### Statistics

Prior to participant recruitment, sample size estimates were calculated with the G*Power software updated in version 3.1.9.4 [54]. Variables were considered as two-tailed and their constant proportionality equal to 0.5. As a good compromise in this kind of research, Type I and II error (α and β levels respectively) were set both [55] at 0.168 [56]. The observed statistical power of the analysis was > 0.8 for all variables and > 0.98 for RPD parameters. Parametric statistics were used after confirming the normal distribution of the dependent variables with the Shapiro-Wilk test. Two methodological approaches were used to verify the hypothesis of the study. First, we used one-way ANOVAs of repeated measures (7x) to compare differences between the basal condition, and the five normalized rounds as well as the recovery. When necessary, the Greenhouse-Geisser’s correction was used if the sphericity test to study matrix proportionality of the dependent variable was significant (p<0.05). If a significant effect was found, a post-hoc analysis was carried out conducting multiple comparisons between the normalized rounds with Sidak adjustment. Eta squared (η^2^) was used to report effect sizes. Second, we used linear regression analysis to study the strength of possible trends between the five normalized rounds of the IFP and the dynamometric parameters. Regression analysis was also used for modeling changes of all variables with the occurrence of fatigue. The level of significance was set at 0.05.

## Results

### Maximal voluntary contraction

**Fig 1:**
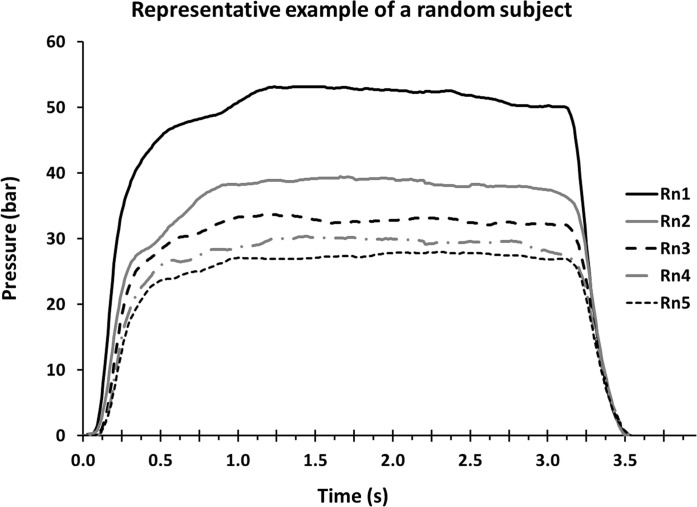
Example of P-t curve from a representative subject during five maximal voluntary contractions (MVC), each corresponding to one of the five normalized rounds (Rn) of the intermittent fatiguing protocol (IFP).

Fig 1 is a representative example of five MVCs obtained from one participant. In comparison to baseline condition, MVC pressure decreased to 62% ±17% of its initial value in the last round of the IFP (*p* < 0.001). Ten minutes after the end of the IFP, MVC pressure was still reduced by 19% ± 14% (*p* < 0.001).

### Kinetics of P-t curve

**Fig 2:**
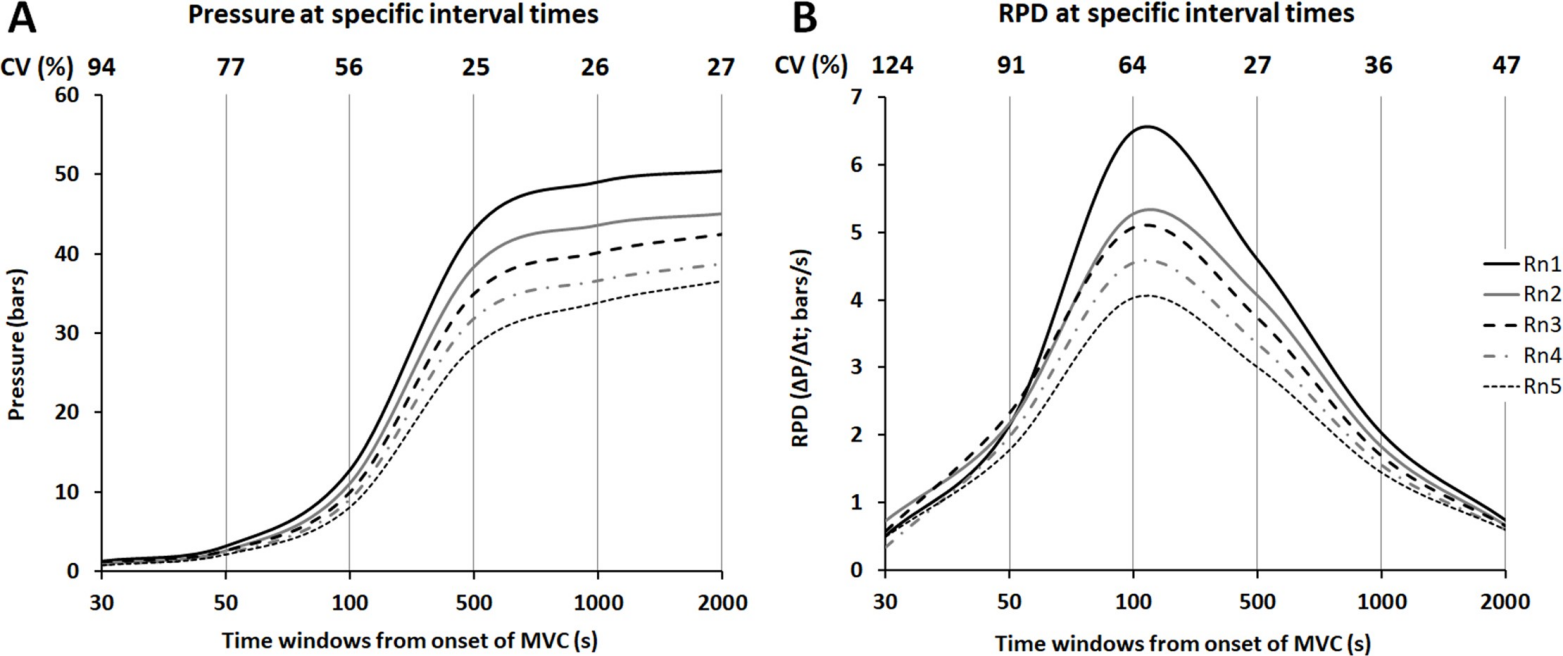
Averaged representation of the pressure (A) and Rate of Pressure Development–RPD- (B) values computed from all participants across specific time intervals in the five normalized rounds (Rn). Presentation of the relative coefficients of variation (CV, %), as representative of the variability between subject, at each time window from the onset of maximal voluntary contraction (MVC).

The decrease of the averaged P-t curve and RPD shapes from all participants throughout the IFP is shown in Fig 2. The decrease is more pronounced in the time window from 500 ms to 1 s for the pressure (Fig 2A) and from 100 ms to 500 ms for the RPD (Fig 2B). It is worth pointing out the very high relative values of the coefficient of variation (between subjects’ analyses of dispersion) during the first 100 ms of MVCs, especially for the RPD (Fig 2B).

**Fig 3:**
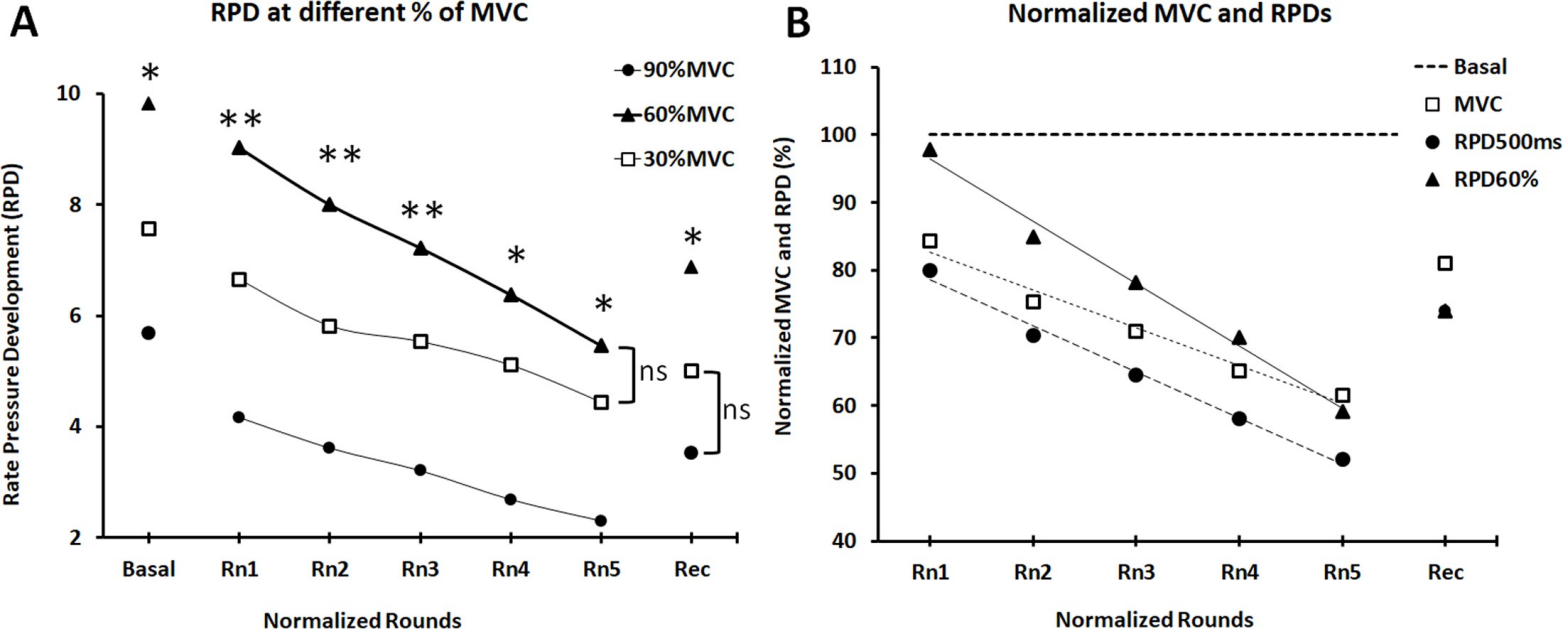
Decrement in the Rate of Pressure Development–RPD- (**Δ**P/**Δ**t) at different MVC intensities (A). Comparison of the slopes’ decrement of the normalized MVC with respect to relative RPD to 500 ms of contraction (RPD_500ms_) and relative RPD to 60% of MVC (RPD_60%MVC_) (B). “Rn” are normalized rounds and “Rec” is the recovery after 10 min from the end of the intermittent fatiguing protocol (IFP).

Overall, greater RPD values were registered at 60%MVC (10.65 < F < 25.81; *p* < 0.001) in comparison with the other two relative contraction intensities (30% and 90% MVC), except in the fifth normalized round where the difference with 30% MVC was not significant (*p* = 0.120) (Fig 3A). No significant difference (P = 0.129) was observed between 30% and 90% MVC in the recovery state (Fig 3A).

At the end of the IFP, RPD over the first 500 ms (RPD_500ms_) and RPD to 60%MVC (RFD_60%MVC_) were 52% ± 14% and 59% ± 24% respectively in comparison to baseline (*p* < 0.001). Ten minutes after the IFP, RPD_500ms_ and RPD_60%MVC_ reached 74% ± 18% (*p* < 0.001) and 72% ± 29% (*p* < 0.003) of baseline values (Fig 3B).

Significant differences were found among the three main types of variables (Area, RPD and Time) independently of %MVCs (Table 1). Concerning the Area, significant decrements were observed at the lower %MVCs (10% and 30%), between the baseline and recovery conditions and the five normalized rounds, but no differences were found across the normalized rounds (Table 2). If the statistical strength and significance of the differences were greater for the RPD at the higher percentages of MVC (60% and 90% MVC), the time needed to reach a specific %MVC was the weakest and the lesser of the three type variables (Table 1). Differences within the IFP’ normalized rounds, as well as between the baseline and the IFP or between the last normalized rounds and the recovery, were confirmed only under the above-mentioned conditions (Table 1).

**Table 1:** One-way ANOVA of repeated measurements, used to compare differences of the raw values among the seven occasions: Baseline (B), five normalized round of the IFP (1, 2, 3, 4, and 5) and the recovery (Rec). The variables used for comparison were: Area, RPD and Time at a specific percentage of the MVC (10%, 30%, 60% and 90%).

Variable	*F*	*P*	η2	*df*	Post-hoc	*P*
Area _10%MVC_	17.5	0.001	0.36	2.01, 62	B > 1, 2, 3, 4, 5	< 0.001
					R > 1, 2, 3, 4, 5	< 0.045
Area _30%MVC_	12.32	0.001	0.28	2.19, 68	B > 1, 2, 3, 4, 5	< 0.01
					Rec > 5, 4, 3	< 0.05
Area _60%MVC_	5.89	0.001	0.16	3.45, 107	B > 1, 2, 3, 4	< 0.006
Area _90%MVC_	3.18	0.046	0.09	2.11, 65	B > 1	< 0.011
RPD _10% MVC_	10.38	< 0.001	0.25	3.81, 118	B > 2, 3, 4, 5, Rec	< 0.020
					1 > 4, 5	< 0.006
RPD _30% MVC_	15.70	< 0.001	0.34	3.14, 97	B > 2, 3 > 6	< 0.007
					R < B, 1	< 0.019
RPD _60% MVC_	21.19	< 0.001	0.41	2.90, 90	B > 2 > 4 > 5	< 0.01
RPD _90% MVC_	18.29	< 0.001	0.37	3.18, 99	B > 1 > 3 > 5	< 0.046
					B > Rec	< 0.001
Time _10%MVC_	5.60	< 0.008	0.15	1.78, 55	4 < Rec	< 0.049
Time _30%MVC_	7.68	< 0.001	0.20	2.40, 74	B < 1, 2, 3, 4	< 0.034
Time _60%MVC_	5.01	< 0.002	0.14	3.17, 98	5 > 1, 2, 3, 4	< 0.039
Time _90%MVC_	2.75	< 0.050	0.08	2.84, 88	5 < 2, 3	< 0.041

Area is computed as time-pressure integral at the specific time-windows of analysis [bar·s]. Rate of Pressure Development (RPD).

**Table 2:** One-way ANOVA of repeated measurements used to compare differences of the normalized values (% of Baseline) among the five normalized rounds of the IFP (1, 2, 3, 4, and 5) and the recovery (Rec). The variables used for comparison were: Area, RPD and time corresponding to a percentage of the MVC (10%, 30%, 60% and 90%).

Variable	*F*	*P*	η2	df	Post-hoc	*P*
nArea _10%MVC_	8.37	< 0.001	0.21	2.36, 73	Rec > 1, 2, 3, 4, 5	< 0.042
nArea _30%MVC_	7.96	< 0.001	0.20	3.03, 94	Rec > 2, 3, 4, 5	< 0.027
nArea _60%MVC_	0.53	0.755	0.09	5, 27		
nArea _90%MVC_	0.55	0.734	0.09	5, 27		
nRPD_10% MVC_	3.80	0.014	0.11	2.91, 90	1 > 3, 4	< 0.003
nRPD_30% MVC_	9.94	< 0.001	0.24	3.04, 94	1 > 4, 5	< 0.001
					Rec < 1	< 0.05
nRPD_60% MVC_	23.54	< 0.001	0.43	2.90, 89	1 < 2 < 4 < 5	< 0.01
					Rec < 1	< 0.001
nRPD_90% MVC_	8.08	< 0.001	0.21	3.22, 100	1 > 3 > 5	< 0.022
					Rec > 5	< 0.03
nTime _10%MVC_	2.22	0.081	0.29	5, 27		
nTime _30%MVC_	1.66	0.177	0.24	5, 27		
nTime _60%MVC_	5.88	< 0.002	0.16	2.47, 77	1, 2, 3 > 5	< 0.05
nTime _90%MVC_	2.40	0.064	0.31	5, 27		

When comparing the normalized variables with respect to basal conditions, it appeared that the time needed to reach a specific %MVC did not increase significantly throughout the IFP, except for 60%MVC (Table 2). With respect to the normalized area, significant differences were observed only between the recovery and the normalized round of the IFP (p < 0.042) at the lower MVC intensities (10%-30%MVC) but not among the normalized round of the IFP. It is remarkable that the greatest decrements were observed in RPDs, especially at 60%MVC (Table 2).

### MVC time-windows of analysis

It is noteworthy that no significant differences were observed at time windows shorter than 100 ms from the onset of the MVC for any of the raw (Table 3) and normalized variables (Table 4). On the contrary, the overall strongest and more significant decrements during MVCs were observed in time windows of 0–500 ms and 0–1 s (Table 4). In particular, this observation is verified with the repeated measures ANOVA in RPDs not only when comparing the baseline against the IFP rounds or the recovery (Table 3), but also among the normalized rounds of the IFP (Table 4).

**Table 3:** One-way ANOVA of repeated measurements used to compare differences of the raw values among the seven occasions: Baseline (B), five normalized round of the IFP (1, 2, 3, 4, and 5) and the recovery (Rec). The variables used for comparison were: Area, RPD and estimated force at the times of 30 ms, 50ms, 100ms, 500 ms, 1 s and 2 s from the onset of the muscular contraction.

Variable	*F*	*P*	η2	*df*	Post-hoc	*P*
Area _30ms_	01.87	0.127	0.31	6, 25		
Area _50ms_	0.94	0.487	0.18	6, 25		
Area _100ms_	2.18	< 0.093	0.07	3.12, 97		
Area _500ms_	47.48	< 0.001	0.61	3.30, 102	B > 1 > 2 > 4 > 5	< 0.006
					B > Rec > 5	< 0.019
Area _1s_	84.36	< 0.001	0.73	3.40, 105	B > 1 > 2 > 3 > 4 > 5	< 0.002
					B > Rec >5, 4	< 0.001
Area _2s_	107.66	< 0.001	0.78	3.33, 103	B > 1 > 2 > 3 > 4 > 5	< 0.008
					B > Rec >5, 4, 3	< 0.001
RPD _30ms_	0.81	0.574	0.16	6, 25		
RPD _50ms_	1.03	0.428	0.20	6, 25		
RPD _100ms_	8.67	< 0.001	0.22	2.77, 86	B > 5, Rec	< 0.021
					1 > 4, 5, Rec	< 0.023
RPD _500ms_	71.57	< 0.001	0.70	3.35, 104	B > 1 > 2 > 4 > 5	< 0.003
					B > Rec > 5, 4	< 0.001
RPD _1s_	70.92	< 0.001	0.70	3.20, 99	B > 1 > 2 > 3 > 4, 5	< 0.024
					B > Rec > 5, 4, 3	< 0.003
RPD _2s_	25.99	< 0.001	0.46	3.07, 95	B > 1 > 2	< 0.013
					B > Rec >5, 4, 3	< 0.042
Pval_30ms_	1.14	0.370	0.21	6, 25		
Pval_50ms_	1.80	0.141	0.30	6, 25		
Pval_100ms_	9.24	< 0.001	0.22	3.24, 103	B > Rec	< 0.011
					1 > 2 > 5	< 0.015
Pval_500ms_	67.03	< 0.001	0.67	2.63, 84	B > 1 > 2 > 3 > 4 > 5	< 0.001
					B > Rec > 5	< 0.002
Pval_1s_	80.51	< 0.001	0.72	2.93, 94	B > 1 > 2 > 3 > 4 > 5	< 0.009
					B > Rec > 5, 4	< 0.002
Pval_2s_	87.14	< 0.001	0.74	2.67, 83	B > 1 > 2 > 3 > 4	< 0.007
					B > Rec > 5, 4, 3	< 0.001

**Table 4:** One-way ANOVA of repeated measurements used to compare differences of the normalized values (% of Baseline) among the five normalized rounds of the IFP (1, 2, 3, 4, and 5) and the recovery (Rec). The variables used for comparison were: Area, RPD and pressure at the times 30 ms, 50 ms, 100 ms, 500 ms, 1 s and 2 s from the onset of the muscular contraction.

Variable	*F*	*P*	η2	df	Post-hoc	*P*
nArea _30ms_	1.20	0.335	0.18	5, 27		
nArea _50ms_	0.99	0.442	0.16	5, 27		
nArea _100ms_	1.39	0.255	0.04	2.28, 71		
nArea _500ms_	28.52	< 0.001	0.48	2.43, 75	1 > 2 > 4 > 5	< 0.001
					R < 1; Rec > 5	< 0.01
nArea _1s_	53.80	< 0.001	0.63	2.95, 91	1 > 2 > 3 > 4 > 5	< 0.002
					R < 1; Rec > 5, 4	< 0.004
nArea _2s_	66.85	< 0.001	0.68	3.13, 2	1 > 2 > 3 > 4 > 5	< 0.003
					Rec < 1	< 0.05
					Rec > 5, 4, 3	< 0.001
nRPD_30ms_	1.80	0.145	0.24	5, 27		
nRPD_50ms_	0.56	0.731	0.94	5, 27		
nRPD_100ms_	8.63	< 0.001	0.22	3.29, 102	1 > 2, 3, 4, 5, Rec	< 0.05
					Rec < 1	< 0.001
nRPD_500ms_	35.43	< 0.001	0.53	2.67, 83	1 > 2 > 3 > 4 > 5	< 0.01
					Rec > 5, 4	< 0.001
nRPD_1s_	27.61	< 0.001	0.47	2.99, 93	1 > 2 > 4	< 0.01
					Rec > 5, 4, 3	< 0.01
nRFD_2s_	11.25	< 0.001	0.27	2.48, 77	1 > 2, 4, 5	< 0.01
					Rec > 5, 4	< 0.002
nPval_30ms_	0.80	0.558	0.13	5, 27		
nPval_50ms_	1.27	0.306	0.19	5, 27		
nPval_100ms_	6.98	< 0.001	0.18	3.57, 111	1 > 4, 5, Rec	< 0.009
nPval_500ms_	47.71	< 0.001	0.61	2.66, 82	1 > 2 > 3 > 4 > 5	< 0.001
					Rec < 1, Rec > 5, 4	< 0.041
nPval_1s_	67.60	< 0.001	0.69	3.03, 94	1 > 2 > 3 > 4 > 5	< 0.006
					Rec > 5, 4, 3	< 0.001
nPval_2s_	51.77	< 0.001	0.63	2.85, 88	1 > 2 > 3 > 4, 5	< 0.004
					Rec > 5, 4, 3	< 0.001

### Regression analysis

**Fig 4:**
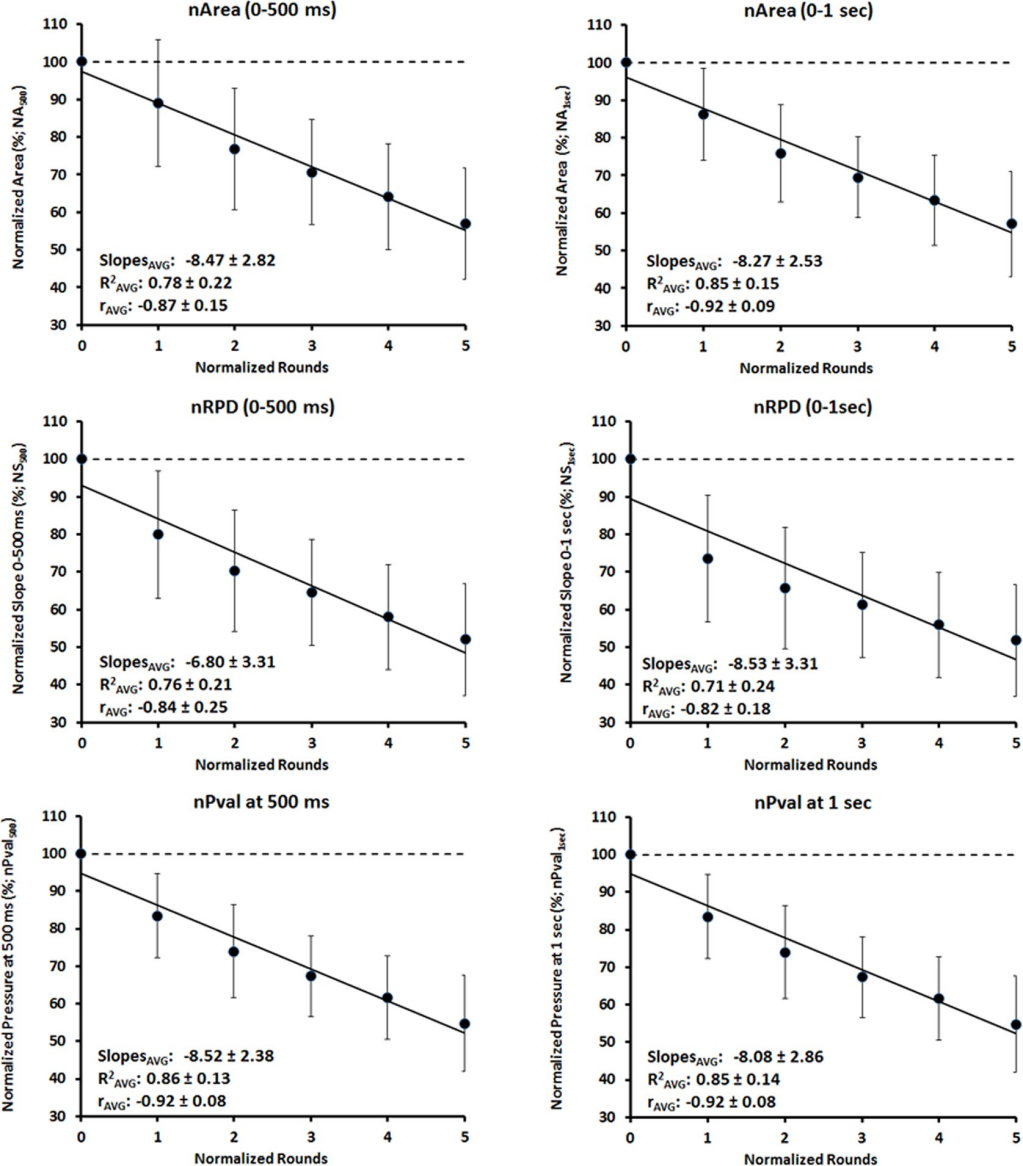
Regression analysis of the normalized area, Rate Pressure Development (RPD), and Pressure value (Pval) with respect to the basal condition, during the intermittent fatiguing protocol (IFP). Time windows from the onset of contraction were 0–500 ms and 0–1 s. Data are mean and standard deviation.

The previous observations are reinforced by the regression analysis performed within the IFP with the area, RPD and force normalized with the basal condition (Fig 3). The strong and significant linear trend (p< 0.001) observed at the time windows 0-500ms (F = 197.83; η^2^ = 0.86) and 0–1 s (F = 139.06; η^2^ = 0.81) support the decline in normalized pressures throughout the IFP particularly at these time intervals of analysis (Fig 4). Similar observations can be highlighted with the linear (P < 0.001) decrement of the RPD (F = 139.37; η^2^ = 81) and the Area (F = 120.12; η^2^ = 0.79) at the time windows of 0–500 ms, as well as at the time window of 0-1s for RFD (F = 69.46; η^2^ = 0.69) and Area (F = 184.75; η^2^ = 0.85) (Fig 4).

The comparison of the linear regression slopes obtained for MVC, RPD_500ms_ and RPD_60%MVC_ during the IFP (Fig 3B) revealed a greater rate of decline for RPD_60%MVC_ in comparison to MVC (P < 0.001) as well as for RPD_60%MVC_ in comparison to RPD500ms (P = 0.044). This observation is strengthened by the significant decrease (P < 0.003) of the ratio RPD_500ms_/MVC (F = 5.28; η^2^ = 0.14) and the ratio RPD_60%MVC_/MVC (F = 5.012; η^2^ = 0.14) throughout the IFP.

## Discussion

This study focused on 30 raw and 30 normalized kinetics-related parameters of the MVC to characterize the P-t curve generated when pulling a lever with two fingers (simulated braking action on a motorcycle), with the objective to determine which ones of them are more relevant and discriminative of a fatigued state. When testing a functional capacity in a specific context, it is much more operative using the lesser possible number of parameters. Therefore, the main objective of this investigation was to give a feedback to the participant about his performance capacity in the most concise and specific manner. Our results did not entirely support the initial hypothesis. The earliest time windows (0–30 ms and 0–50 ms) and the lower intensity of contraction (10% MVC) of the P-t curve were neither sensitive enough, nor useful to assess neuromuscular fatigue when braking repeatedly on a setup replicating the actions on a motorcycle.

It is noteworthy that fatigue-related decrements of braking performance were observed only at time intervals longer than 100 ms and contraction intensities greater than 30%MVC.

Before interpreting and comparing our results with the literature, it is essential to keep in mind the mechanical compliance of the braking system of a motorcycle (see instrumental setup). The compliance of the system is of utmost importance to properly interpret our RPD and especially the early phase of the P-t curves [57]. Usually, instrumental setup is as rigid as possible with the agonist muscle placed under a slight stretch [57]. In the present study, we decided to replicate in the field conditions in order to facilitate the applicability of the information to technical staff, coaches and motorcycle riders. As a consequence of our approach, longer time is needed to obtain initial increases of pressure inside the hydraulic braking system. Moreover, when the testing movement is of multi-joint nature, as in our study, it is well accepted that the experimental control of the assessment is more difficult during the shorter time-windows of the P-t curve [23, 24, 36]. In single joint movements (elbow flexion or knee extensors), the individual can reach high MVC levels (around 70% MVC) in short-time intervals (~150 ms) in a non-fatigued state [48, 49]. But, when comparing single-joint with multi-joint movements such as handgrip actions, the overall times to develop force are longer for the second ones. This observation is supported by the fact that the time needed for our subjects to reach 60% MVC in basal conditions was 0.28±0.14 s. Again, before comparing handgrip tests of different nature, time differences among studies may be explained by the different characteristics of the instrumental setup, such as the hand and forearm position and the mechanical compliance of each system.

After these methodological considerations, it seems obvious that we cannot adopt the same time criteria proposed by previous studies to differentiate the “early phase” from the “late phase” of our RPD analysis. Moreover, a strict consensus does not exist when it comes to marking off specific time intervals of analysis. From shorter to longer, time-windows of 0–50 ms or shorter than 80 ms [25, 58], 0–100 ms [47], 0–150 ms or 0–200 ms [23, 48], were considered to characterize the “early phase” of RFD. Such suggestions mean that longer time intervals are more appropriate to characterize the “late phase” as long as an ascending part in the F-t curve can still be observed. In the present study we did not obtain consistent and discriminative trends of a loss of performance at time intervals shorter than 500 ms. As a result, in spite of the shorter time-windows used to report the early phase of RFD (≤100 ms) in numerous studies [23, 25, 47, 48], due to the mechanical compliance of a motorcycle hydraulic braking system, we suggest considering the time-window of 0–500 ms, or at least not shorter than ~ 300 ms, as the more appropriate to assess fatigue-related changes of the early phase of RPD. Certainly, a stiffer setup, less similar to the real hydraulic braking systems habitually used nowadays, should shorten our proposed discriminative time-windows of analysis.

On the other hand, the large variability within and between subjects, could explain the absence of significant differences or trends during the first 50 ms. The widely reported variability of these kinds of recording, is mainly due to the great inter-individual variability in the magnitude of muscle activation during MVCs and rapid contractions [24, 58]. The efferent drive to the agonist and antagonist muscles involved varies substantially among subjects, particularly during the early phase of the F-t curve, and reinforce the rationale that, in this phase, neural factors contribute substantially to the variability between subjects [39, 49].

It is well documented that maximal RFD decreases during fatiguing protocols, especially when maximal strength exertions are involved [3, 5, 43]. Our results confirm that the RPD and the shape of the MVC’s P-t curves (areas, RPDs and pressure values at different time-windows) change during our fatiguing protocol, whereas time to reach a determined percentage of MVC lengthens. Our RPD decrement (50% and40% for Pval_1s_ and Pval_2s_, respectively) were very similar to those reported for RFDs up to 50% MVC in Royce’s study [5], who used a fatiguing test consisting in sustaining a MVC. Whereas Ewing and Alan Stull [3] found the time taken to reach 50% MVC after a handgrip fatiguing protocol to be 32% longer with respect to baseline values, in our study the time taken to reach 60% MVC was only 13% longer. Nevertheless, in their study, fatigue induced an RFD decrement of 53% quite similar to our results (decline of 60% and 48% for the RPD_60%MVC_ and RPD_90%MVC_, respectively). Finally, Miller et al. [59] also studied RFD of human hand muscle (first dorsal interosseous), but the adduction gesture of the thumb, the small forces exerted (less than ~40N), and the compliance of the system (considered as virtually isometric with 1mm movement at 100 N force) does not allow direct comparisons with our results. It is clear that when it comes to compare time intervals of the RFD, the compliances of the mechanical setup and the human limb play both a fundamental role, during the assessment of isometric contractions.

Usually, in fast MVCs, the muscle does not reach its maximal RFD capacity due to limitations imposed by neural factors (e.g. rate coding) [60]. This rational is supported by the decline in the slope of the force-time relationship, as muscle force increases [59], particularly from the 60–80% MVC onwards [23]. Our result partially confirmed previous studies as RPD_60%MVC_ was consistently higher than RPD_90%MVC_ and RPD_30%MVC_ independently of the IFP’s normalized round. Considering the overall literature, higher values for RPD at lower percentages of MVC would have been expected. Again, we think that the compliance of our instrumental setup may explain why RPD_30%MVC_ was lower than RPD_60%MVC_, and suggest that 60% MVC is an appropiate relative intensity to obtain the highest RPD when simulating the braking gesture on a motorcycle.

The rate of increase in neural drive (estimated as the average number of motor unit discharges per second) to muscle have a greater influence on RFD [39], particularly in muscles that have a greater proportion of high-threshold motor units containing fast muscle fibers [25]. During the early phase of explosive contractions there is a compression of the recruitment range of motor units that make the high-threshold motor units activated at a lower force level [61], while preserving the recruitment order among motor units (Henneman’s size principle [62]).

When exerting voluntary contractions with the intention of reaching the highest force as fast as possible, the central nervous system generates motor unit discharges at frequencies that are significantly greater compared to sustained MVC [62]. Because a linear relationship exists between the force generated and the RFD as the activation intensity increases [59], we could assume that, with the occurrence of fatigue, the decrease of these variables may be due, at least partially, to a reduction in voluntary activation. This decrease in the early part of the rising phase of voluntary contraction may be primarily due to fatigue-related mechanisms of a central origin [43]. The decrease of the normalized RPD_500ms_ and RPD_60%MVC_ to MVC (ratios RPD_500ms_/MVC and RPD_60%MVC_/MVC) support the idea that when performing the IFP, the participants were losing their ability to develop their available force rapidly, and were, for instance, affected to a certain degree by neural limitations. Unfortunately, it is not possible from our study to determine the underlying mechanisms [40].

### Limitations

As potential limitation of this study first, we did not compare pressure data recorded with an hydraulic device to those obtained with strain-gauge transducers in different setup conditions with the same subjects. A comparison between our relatively compliant system (identical to field conditions) with a more “classical” rigid or quasi-rigid setup (less ecological to riding conditions) would be helpful. Second, as potential confounding factors, it would be advisable to address the reliability aspects of the braking gesture, such as minimal detectable change, with a greater number of participants. By doing this, we would be able to determine exactly how much difference is necessary for it to be considered “real” with the parameters proposed as the most sensitive of a state of fatigue in the present study. Third, our road motorcycle simulator reflects only partially the total force requirements involved in real piloting because it cannot reproduce the frequent and radical tilt changes that generate the inertial forces and angular momentums typically present in real conditions [29].

## Practical applications

After reviewing the literature, we are not aware of any investigation that has implemented the braking gesture on a handlebar similar to those used on a motorcycle to assess RPD and P-t curve characteristics.

In the present study, a great number of variables were initially used to study the P-t curve characteristics during the braking gesture of a motorcycle. As general advice, we suggest using time windows of 0–500 ms, preferably not shorter than ~300 ms to assess changes in the early phase of the maximal voluntary braking force. For the later phase of contraction, the time-window of 0-1s proved also to be useful and discriminative of a fatiguing state.

Moreover, taking into account the submaximal nature of the braking gesture when riding in real conditions, we suggest contraction intensities comprised between 30% and 60% of MVC, as more suitable criteria to study fatigue-related decrements in performance instead of the classical MVC force.

The time necessary to obtain a specific percentage of MVC was the weakest factor to determine the level of neuromuscular fatigability. By using the first two criteria (time interval and %MVC) we consider this third criterion to be “expendable”.

Pressure, RPD (slope) and Area (representative of the impulse), have a very similar time course of change with the occurrence of neuromuscular fatigue. However, we suggest using RPD for its extensive use in related investigations, and Area for its conceptual and interpretative meaning.

## Supporting information

S1 Fig Motorcycle setup. (TIF)

S2 Fig Structure of the intermittent fatiguing protocol (IFP). (TIF)

S3 Fig Parameters used in MVCs. (TIF)

S4 Fig Sequence of a round with raw signal. (TIF)

S1 Dataset Raw data in SPSS data format. (SAV)

S2 Dataset Normalized data in SPSS data format. (SAV)
