# Supplementary figures and images for: Forearm muscles fatigue induced by repetitive braking on a motorcycle is best discriminated by specific kinetic parameters
Source: PLoS One. 2021 Feb 5;16(2):e0246242. doi: 10.1371/journal.pone.0246242 (PMC7864446; doi:10.1371/journal.pone.0246242)

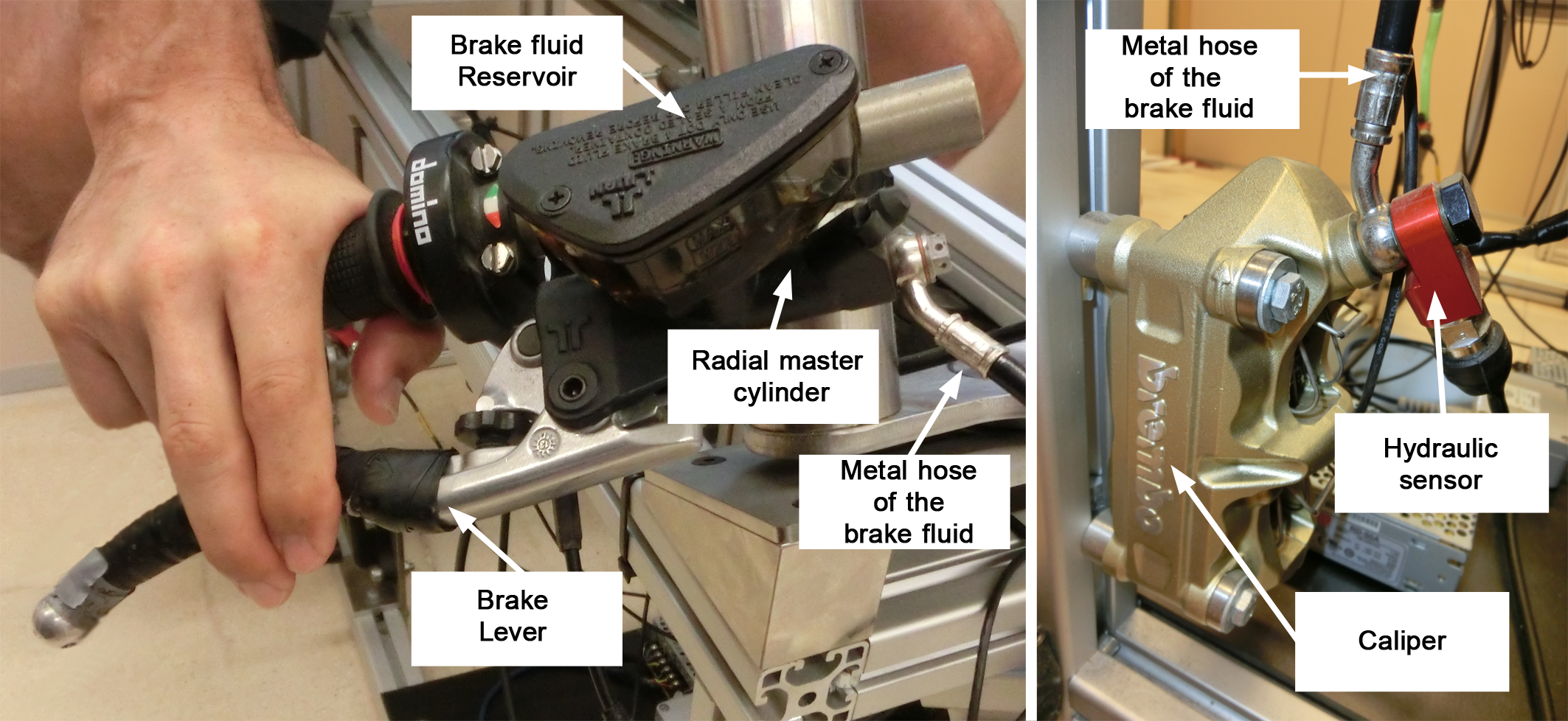

Supplement: S1 Fig — (TIF) [file pone.0246242.s001.tif]

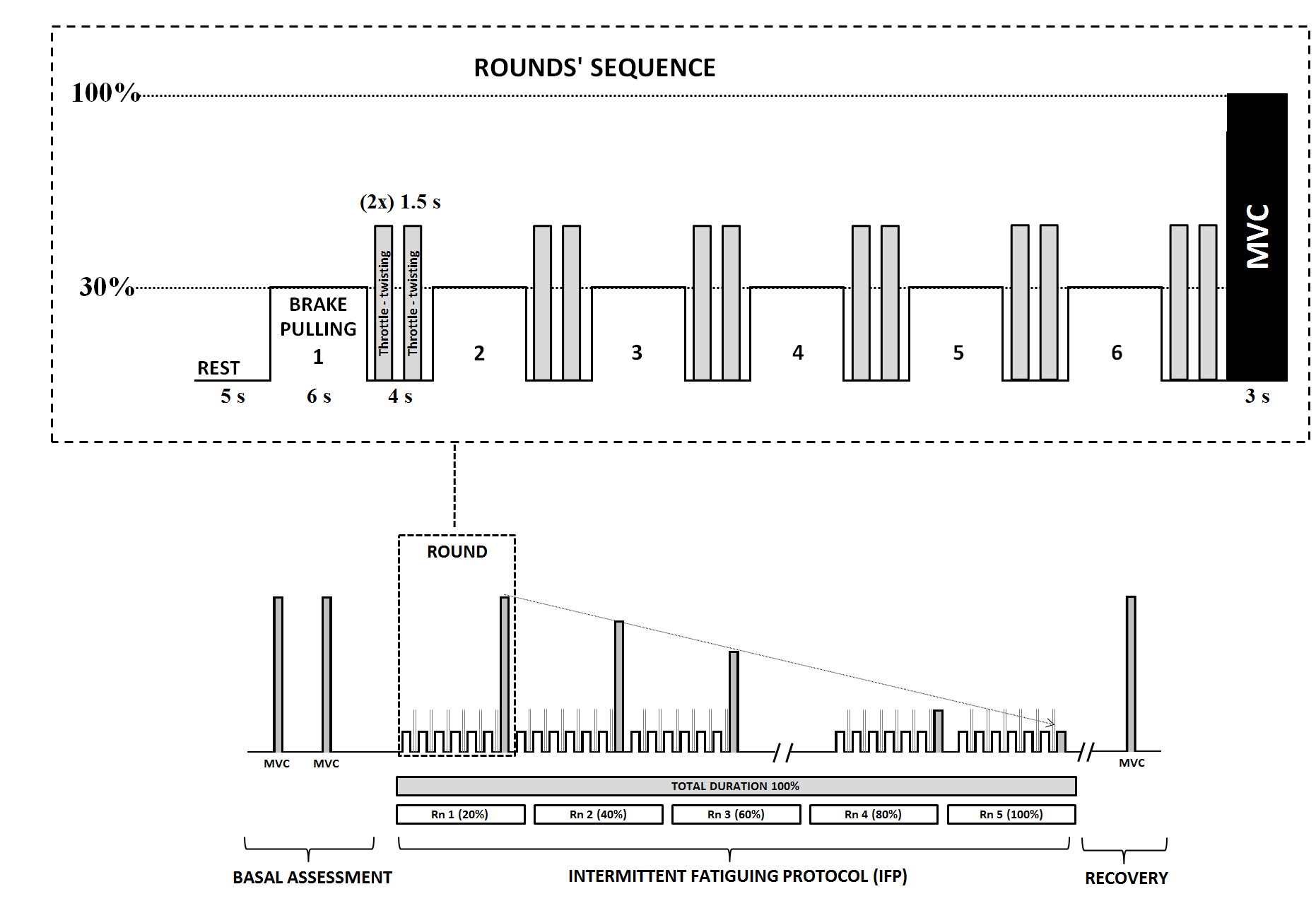

Supplement: S2 Fig — (TIF) [file pone.0246242.s002.tif]

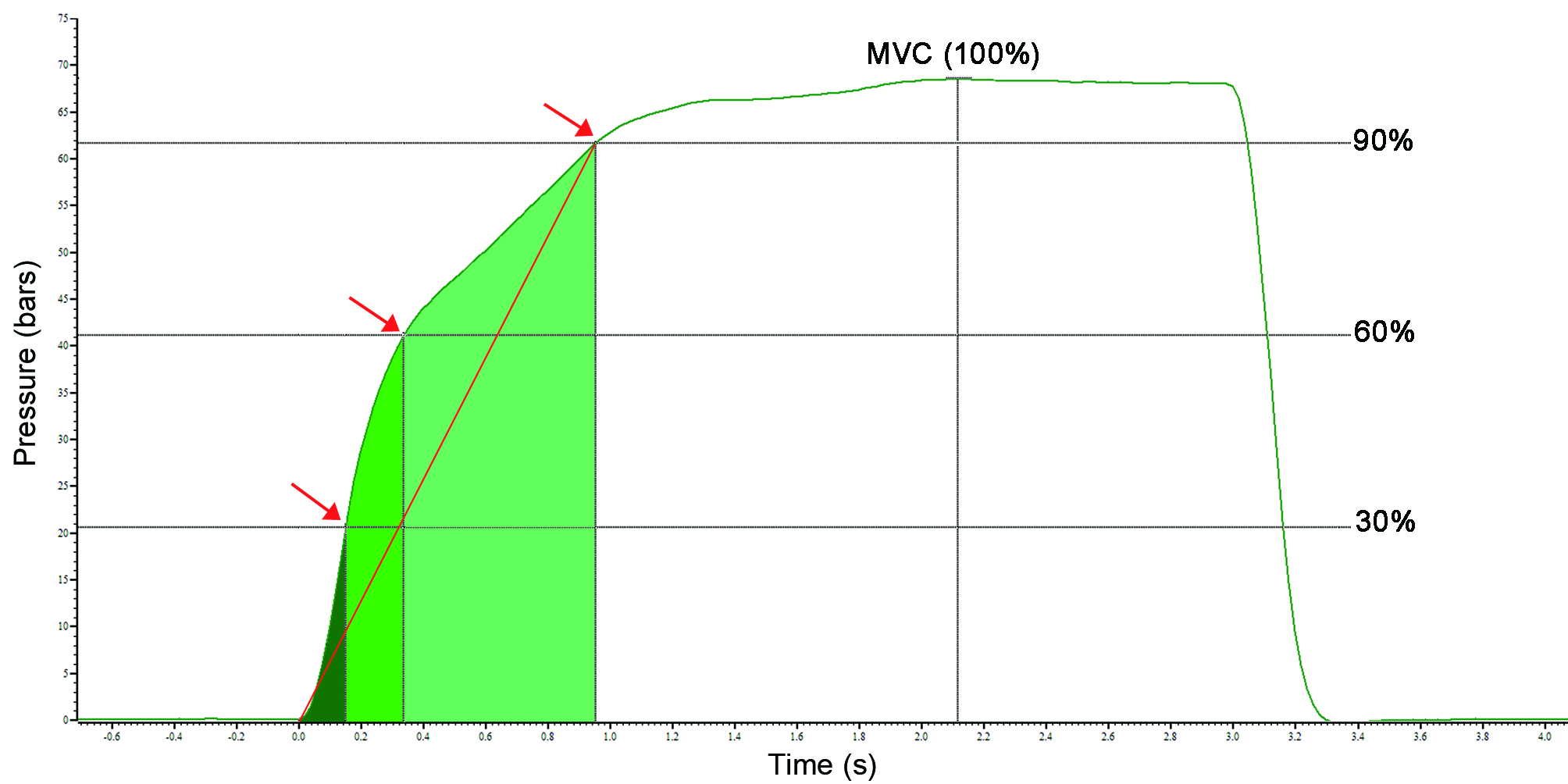

Supplement: S3 Fig — (TIF) [file pone.0246242.s003.tif]

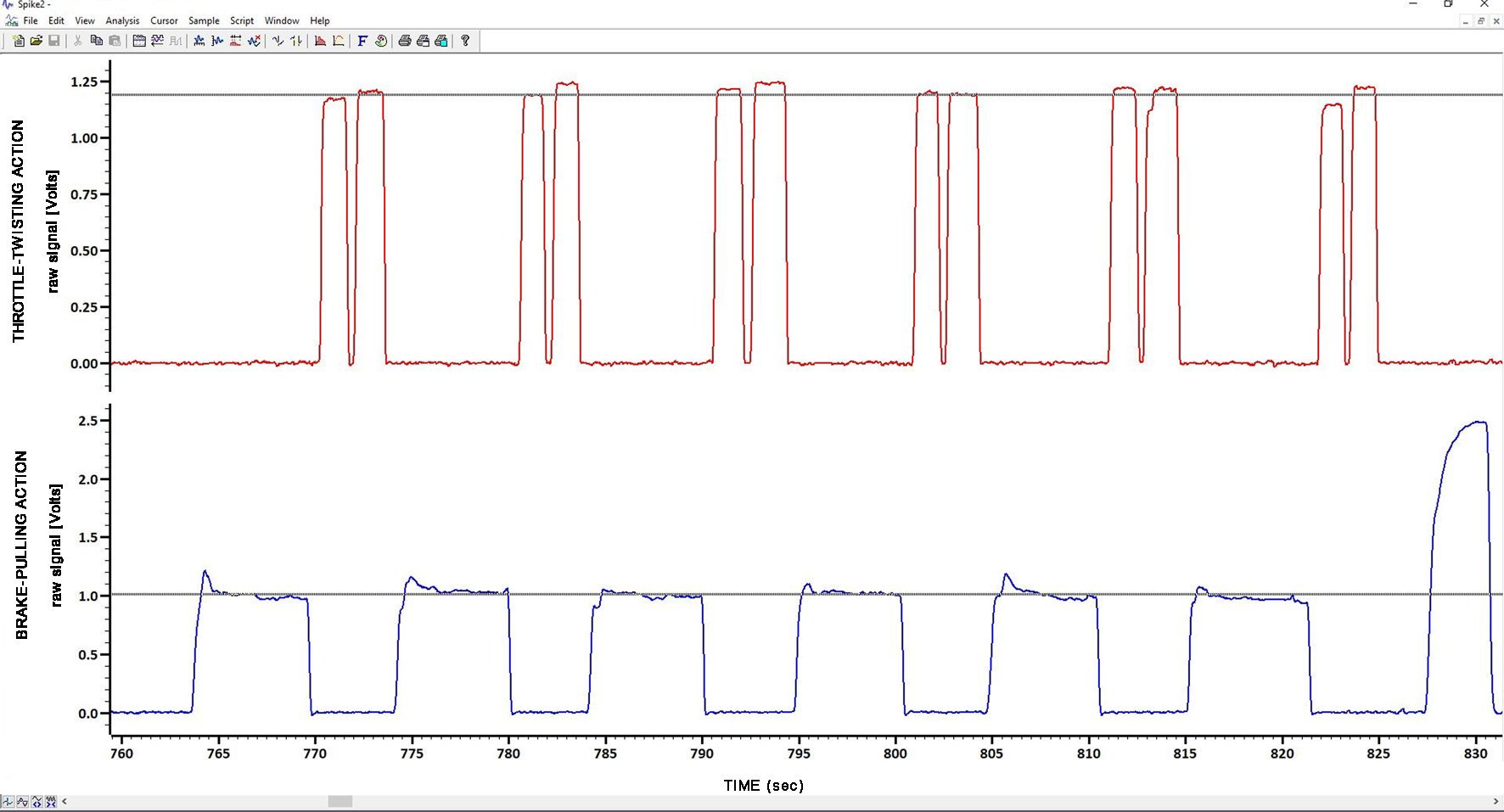

Supplement: S4 Fig — (TIF) [file pone.0246242.s004.tif]
